# First confirmed record of *Buforubroventromaculatus* Orlov, Ananjeva, Ermakov, Lukonina, Ninh & Nguyen, 2024 (Anura, Bufonidae) from China, with supplementary description of this species

**DOI:** 10.3897/BDJ.12.e134392

**Published:** 2024-10-14

**Authors:** Shuo Liu, Mian Hou, Mingzhong Mo, Dingqi Rao

**Affiliations:** 1 Kunming Institute of Zoology, Chinese Academy of Sciences, Kunming, China Kunming Institute of Zoology, Chinese Academy of Sciences Kunming China; 2 Sichuan Normal University, Chengdu, China Sichuan Normal University Chengdu China; 3 Honghe Prefecture Forestry and Grassland Bureau of Yunnan Province, Mengzi, China Honghe Prefecture Forestry and Grassland Bureau of Yunnan Province Mengzi China

**Keywords:** distribution, Guangxi, morphology, ND2, Yunnan

## Abstract

**Background:**

*Buforubroventromaculatus* Orlov, Ananjeva, Ermakov, Lukonina, Ninh & Nguyen, 2024 is a species recently described from Vietnam. Currently, this species is known from central and northern Vietnam and it uncertain whether this species is distributed in China. In addition, the original description of this species is very brief.

**New information:**

Based on nine specimens collected from Yunnan Province and Guangxi Autonomous Region, China, we provide the first confirmed record of *Buforubroventromaculatus* from China. The morphological characteristics of the specimens from China mostly agree with the original description of *B.rubroventromaculatus* and, phylogenetically, the specimens from China clustered with the type series of *B.rubroventromaculatus* from Vietnam. We also provide a supplementary description of this species, based on the specimens we collected.

## Introduction

The genus *Bufo* Garsault, 1764 is a type of large and common toad which is widespread from temperate Eurasia and adjacent islands and Japan south to North Africa, the Middle East, north-eastern and western Myanmar, China and northern Vietnam (Frost 2024). According to Frost (2024), this genus currently contains 26 species, of which 19 are distributed in China.

*Buforubroventromaculatus* Orlov, Ananjeva, Ermakov, Lukonina, Ninh & Nguyen, 2024 was recently described from Ha Tinh Province, Vietnam (Orlov et al. 2024). Currently, *B.rubroventromaculatus* is confirmed to occur in central and northern Vietnam (Orlov et al. 2024). However, in the phylogenetic analysis of Orlov et al. (2024), a sequence (GenBank accession AY936852) of a specimen from Jiangkou County, Guizhou Province, China was clustered with the sequences of *B.rubroventromaculatus*. Since Jiangkou is far away from the type locality (approximately 1150 km) of *B.rubroventromaculatus* and Orlov et al. (2024) did not examine the specimen corresponding to this sequence, the distribution of *B.rubroventromaculatus* in Jiangkou is doubtful and Frost (2024) did not include China in the distribution of this species.

During our herpetological expeditions in Yunnan Province and Guangxi Autonomous Region, China, from 2019 to 2021, we collected some specimens of *Bufo* that were considered *B.gargarizans* at that time. After re-examination of these specimens, we found that they should be assigned to the recently described species *B.rubroventromaculatus*. Herein, we confirm the distribution of *B.rubroventromaculatus* in China and provide a supplementary morphological description of this species, based on the specimens collected from China.

## Materials and methods

**Figure 1.**
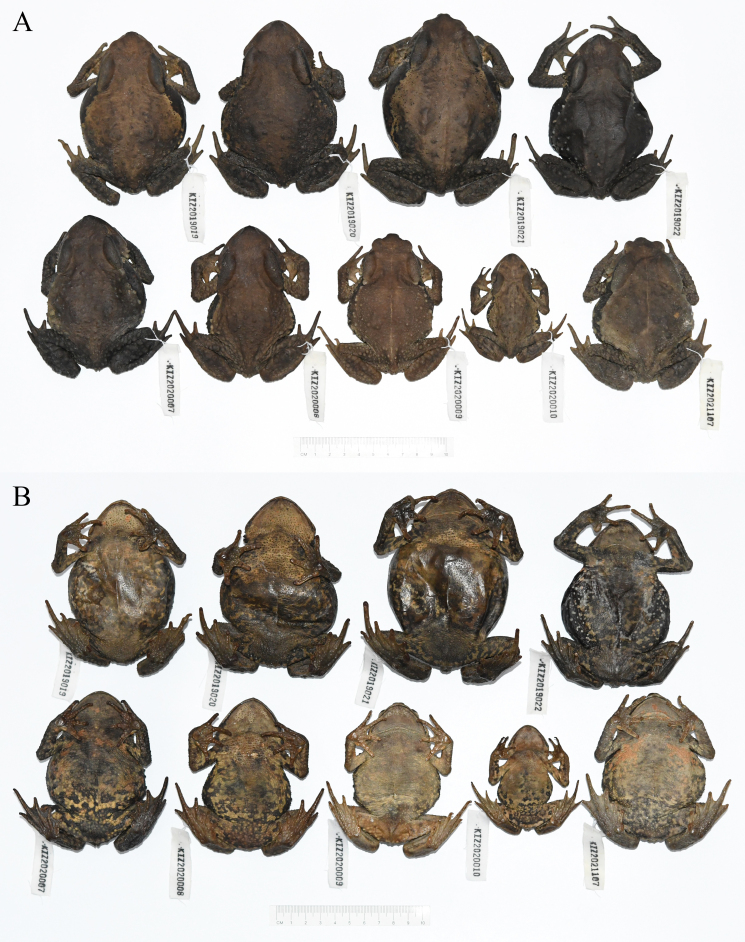
Specimens of *Buforubroventromaculatus* from China in preservative. **A** Dorsal view; **B** ventral view.

Specimens were collected by hand. Liver tissues were stored in analytical pure ethanol and toads were preserved in 75% ethanol. All specimens (Fig. 1) were deposited at Kunming Natural History Museum of Zoology, Kunming Institute of Zoology, Chinese Academy of Sciences (KIZ).

Measurements were taken with a digital caliper to the nearest 0.1 mm. The methodology of measurements followed Orlov et al. (2024). The following morphological characteristics were noted: snout-vent length (SVL); head length (HL), from the back of the mandible to the tip of the snout; head width (HW), across the angles of the jaws; snout length (SNL), from the front of the eye to the tip of the snout; distance from the back of the mandible to the nostril (MN); distance from the back of the mandible to the front of the eye (MFE); distance from the back of the mandible to the back of the eye (MBE); horizontal eye diameter (ED); internasal distance (IN); distance between the front of the eyes (DAE); distance between the back of the eyes (DPE); nostril–snout distance (NS); eye–nostril distance (EN); distance from the axilla to the elbow (FLL); distance from the elbow to the tip of Finger III (HAL); inner palmar tubercle length (IPT); outer palmar tubercle length (OPT); finger I length (F1); finger II length (F2); finger III length (F3); and finger IV length (F4).

A fragment of the mitochondrial NADH dehydrogenase subunit 2 gene (ND2) was amplified via the polymerase chain reaction (PCR) using the primers L-int: 5′-AGC ATC CTA CCC ACG ATT TCG-3′ (Fu et al. 2005) and H4980: 5′-ACT TTT CGG ATT TGA GTT TGG TT-3′ (Macey et al. 1998). The experimental protocols for amplification and sequencing followed Orlov et al. (2024). Sequences were stitched using SeqMan in Lasergene 7.1 (Burland 2000). All new sequences have been deposited in GenBank under the accessions PQ404040–PQ404048.

Sequences were aligned using ClustalW (Thompson et al. 2002) with default parameters in MEGA 11 (Tamura et al. 2021). Uncorrected pairwise distances were calculated in MEGA 11. The best substitution model was selected using the corrected Bayesian Information Criterion (BIC) in ModelFinder (Kalyaanamoorthy et al. 2017). Bayesian Inference was performed in MrBayes v.3.2.7 (Ronquist et al. 2012) under the selected substitution model (GTR+F+I+G4). The Markov chains were run for 5,000,000 generations and sampled every 100 generations. Maximum Likelihood analysis was conducted in raxmlGUI v.2.0 (Edler et al. 2021) under the selected substitution model (TN+F+I+G4) with 1,000 ultrafast bootstrap replicates.

## Taxon treatments

### Bufo rubroventromaculatus

Orlov, Ananjeva, Ermakov, Lukonina, Ninh & Nguyen, 2024

3ED0E6EC-23EE-5030-B36C-1E5D27BB138C

#### Materials

- **Type status:** Other material. **Occurrence:** catalogNumber: KIZ2019019; individualCount: 1; sex: female; lifeStage: adult; occurrenceID: 6429884F-DE2B-5307-A1C1-2FF99671A6BC; **Taxon:** scientificName: *Buforubroventromaculatus*; **Location:** country: China; stateProvince: Guangxi; locality: Bainan Township, Napo County, Baise City; verbatimElevation: 1030 m; verbatimCoordinates: 23°1'20"N 105°50'58"E; **Event:** eventRemarks: collected by Shuo Liu on 25 March 2019; **Record Level:** basisOfRecord: preserved specime
- **Type status:** Other material. **Occurrence:** catalogNumber: KIZ2019020; individualCount: 1; sex: female; lifeStage: adult; occurrenceID: AF1C5F73-C95C-576F-A98D-99E3714C3933; **Taxon:** scientificName: *Buforubroventromaculatus*; **Location:** country: China; stateProvince: Guangxi; locality: Bainan Township, Napo County, Baise City; verbatimElevation: 1030 m; verbatimCoordinates: 23°1'20"N 105°50'58"E; **Event:** eventRemarks: collected by Shuo Liu on 25 March 2019; **Record Level:** basisOfRecord: preserved specime
- **Type status:** Other material. **Occurrence:** catalogNumber: KIZ2019021; individualCount: 1; sex: female; lifeStage: adult; occurrenceID: 68661B2A-10DB-54AC-9FA8-A4BE58F877E3; **Taxon:** scientificName: *Buforubroventromaculatus*; **Location:** country: China; stateProvince: Guangxi; locality: Bainan Township, Napo County, Baise City; verbatimElevation: 1030 m; verbatimCoordinates: 23°1'20"N 105°50'58"E; **Event:** eventRemarks: collected by Shuo Liu on 25 March 2019; **Record Level:** basisOfRecord: preserved specime
- **Type status:** Other material. **Occurrence:** catalogNumber: KIZ2019022; individualCount: 1; sex: female; lifeStage: adult; occurrenceID: C597E27A-BB8C-5EB5-8B52-10C52C9853B1; **Taxon:** scientificName: *Buforubroventromaculatus*; **Location:** country: China; stateProvince: Guangxi; locality: Bainan Township, Napo County, Baise City; verbatimElevation: 1030 m; verbatimCoordinates: 23°1'20"N 105°50'58"E; **Event:** eventRemarks: collected by Shuo Liu on 25 March 2019; **Record Level:** basisOfRecord: preserved specime
- **Type status:** Other material. **Occurrence:** catalogNumber: KIZ2020007; individualCount: 1; sex: female; lifeStage: adult; occurrenceID: 6341C8F5-979F-5396-9FC0-2923CD457CB0; **Taxon:** scientificName: *Buforubroventromaculatus*; **Location:** country: China; stateProvince: Yunnan; locality: Xiajinchang Township, Malipo County, Wenshan Prefecture; verbatimElevation: 1550 m; verbatimCoordinates: 23°12'12"N 104°47'12"E; **Event:** eventRemarks: collected by Shuo Liu on 23 July 2020; **Record Level:** basisOfRecord: preserved specime
- **Type status:** Other material. **Occurrence:** catalogNumber: KIZ2020008; individualCount: 1; sex: female; lifeStage: adult; occurrenceID: EB2A98E9-C0C2-54AE-94F1-AE2151C729B6; **Taxon:** scientificName: *Buforubroventromaculatus*; **Location:** country: China; stateProvince: Yunnan; locality: Xiajinchang Township, Malipo County, Wenshan Prefecture; verbatimElevation: 1550 m; verbatimCoordinates: 23°12'12"N 104°47'12"E; **Event:** eventRemarks: collected by Shuo Liu on 23 July 2020; **Record Level:** basisOfRecord: preserved specime
- **Type status:** Other material. **Occurrence:** catalogNumber: KIZ2020009; individualCount: 1; sex: male; lifeStage: adult; occurrenceID: 950E6AAA-E958-5611-951D-B74AB0312E98; **Taxon:** scientificName: *Buforubroventromaculatus*; **Location:** country: China; stateProvince: Yunnan; locality: Xiajinchang Township, Malipo County, Wenshan Prefecture; verbatimElevation: 1550 m; verbatimCoordinates: 23°12'12"N 104°47'12"E; **Event:** eventRemarks: collected by Shuo Liu on 23 July 2020; **Record Level:** basisOfRecord: preserved specime
- **Type status:** Other material. **Occurrence:** catalogNumber: KIZ2020010; individualCount: 1; sex: female; lifeStage: adult; occurrenceID: C8294D88-326F-57AF-B607-B4BA846C3FE2; **Taxon:** scientificName: *Buforubroventromaculatus*; **Location:** country: China; stateProvince: Yunnan; locality: Xiajinchang Township, Malipo County, Wenshan Prefecture; verbatimElevation: 1550 m; verbatimCoordinates: 23°12'12"N 104°47'12"E; **Event:** eventRemarks: collected by Shuo Liu on 23 July 2020; **Record Level:** basisOfRecord: preserved specime
- **Type status:** Other material. **Occurrence:** catalogNumber: KIZ2021107; occurrenceID: DEA27300-CD84-5F69-B48E-6A062DFE0889; **Taxon:** scientificName: *Buforubroventromaculatus*; **Location:** country: China; stateProvince: Yunnan; locality: Xiajinchang Township, Malipo County, Wenshan Prefecture; verbatimElevation: 1780 m; verbatimCoordinates: 23°7'2"N 104°50'7"E; **Event:** eventRemarks: collected by Shuo Liu on 10 May 2021

#### Description of the specimens from China

Male body size relatively small, SVL 71.6 mm, female body size large, SVL 95.5–123.7 mm; head relatively large (HL/SVL 0.32–0.35, HW/SVL 0.40–0.45), wider than long (HW/HL 1.17–1.35); snout obtuse, relatively short (SNL/SVL 0.12–0.14), greater than eye horizontal diameter (ED/SNL 0.61–0.86); canthus rostralis distinct; loreal region and oblique; nostril to snout tip distance smaller than or subequal to eye to nostril distance (NS/EN 0.86–1.05); pupil horizontal; tympanum distinct; vomerine teeth present; tongue not notched posteriorly.

Female fore-limb relatively long (FLL/SVL 0.18–0.24, HAL/SVL 0.49–0.54), male fore-limb quite long (FLL/SVL 0.27, HAL/SVL 0.57); relative length of fingers F3 > F1 > F4 > F2 or F3 > F4 > F1 > F2; webbing between fingers absent; subarticular tubercles and supernumerary tubercles present; inner palmar tubercle small, oval; outer palmar tubercle large, round; nuptial pad present on first and second fingers in adult male.

Hind-limb relatively short; relative length of toes T4 > T5 > T3 > T2 > T1; webbing between toes present and undeveloped, approximately half-webbed; subarticular tubercles present; inner metatarsal tubercle developed, oval; outer metatarsal tubercle present.

Dorsal surface of head smooth with few tubercles; dorsal and lateral surface of body with many small to medium-sized tubercles; parotoid glands developed and large; dorsolateral fold absent; dorsal surface of fore-limbs with many small to medium-sized tubercles, dorsal surface of hind-limbs with many small to large-sized tubercles; ventral surface relatively smooth with tiny tubercles.

#### Colouration in life

**Figure 2.**
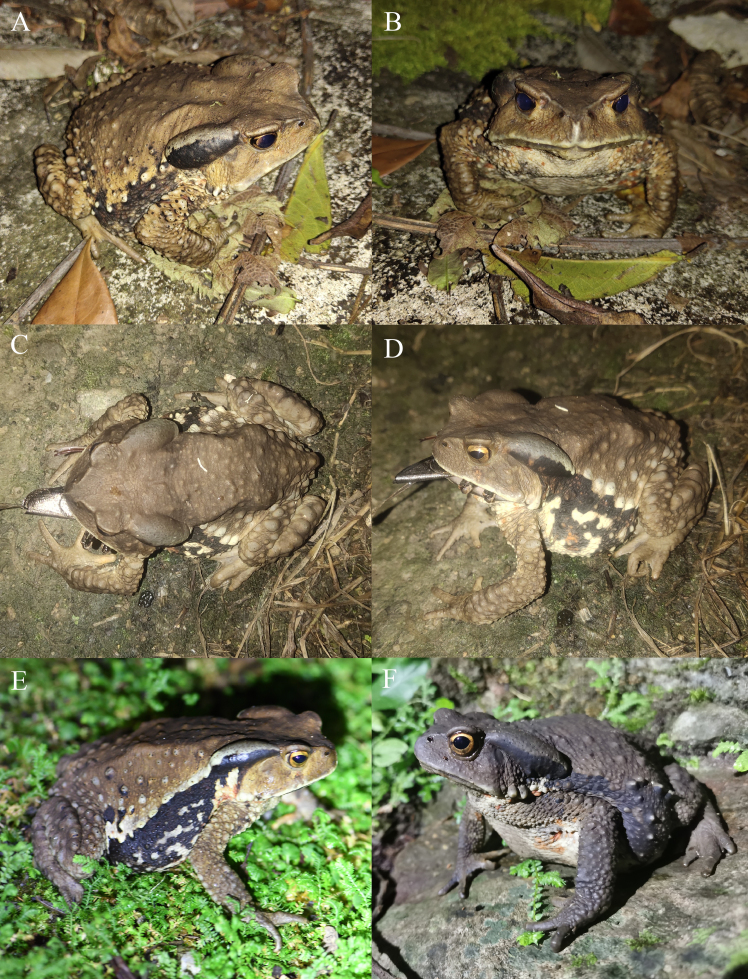
*Buforubroventromaculatus* from China in life. **A, B** The female (KIZ2019020) from Bainan Township, Napo County, Baise City, Guangxi Autonomous Region; **C, D** an uncollected female from Xiajinchang Township, Malipo County, Wenshan Prefecture, Yunnan Province; **E, F** two uncollected females from Tianbao Town, Malipo County, Wenshan Prefecture, Yunnan Province.

Dorsal surface yellowish-brown, brown or blackish-brown; a black stripe from ventral parotoid gland to groin region on each side, with white upper edge in some individuals; lower flanks mottled with black, white and a little red; ventral surface yellowish-white or greyish-white, anterior region with some red spots, posterior region with many black blotches; iris golden (Fig. 2).

#### Distribution

**Figure 3.**
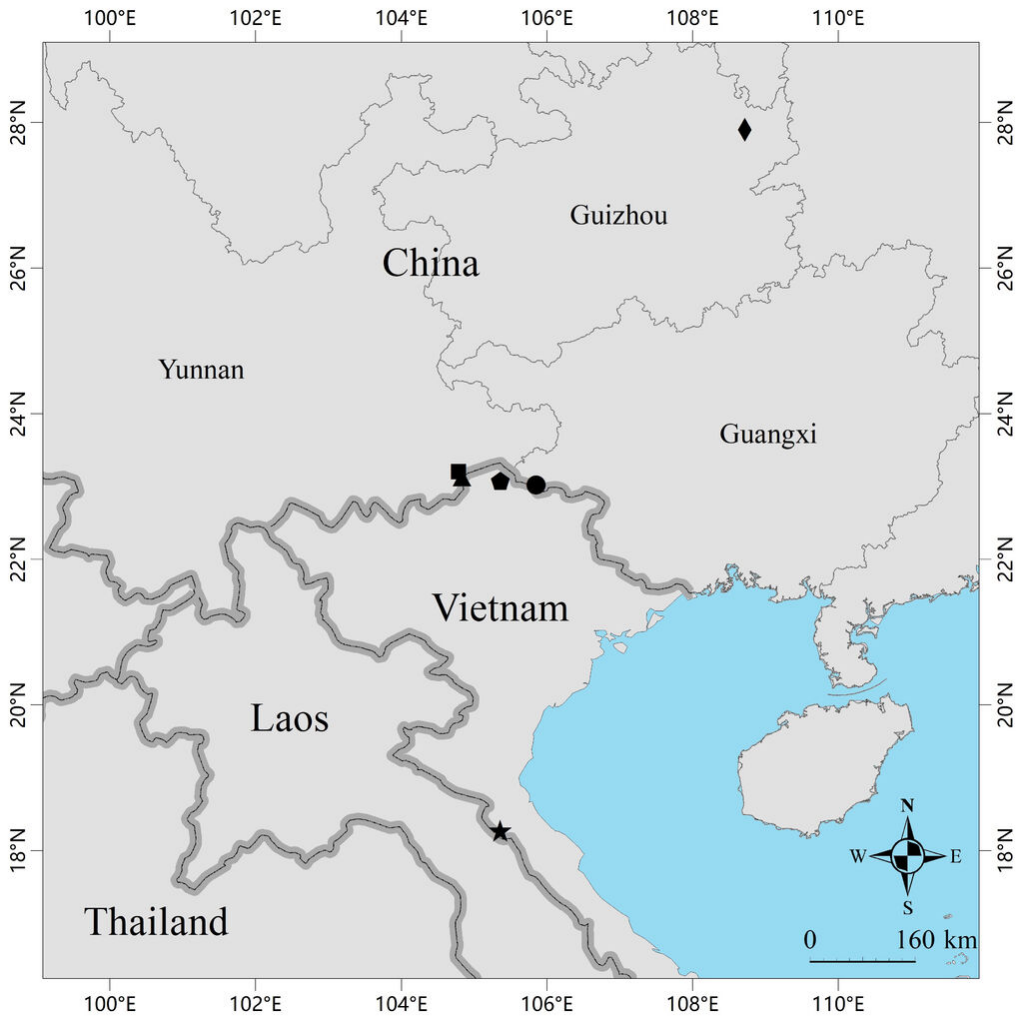
Map showing the type locality of *Buforubroventromaculatus* in Ha Tinh Province, Vietnam (black star), the collection site of the paratypes of *B.rubroventromaculatus* in Ha Giang Province, Vietnam (black pentagon), the collection sites of the specimens from Xiajinchang Township, Malipo County, Wenshan Prefecture, Yunnan Province, China (black square and black triangle), the collection site of the specimens from Bainan Township, Napo County, Baise City, Guangxi Autonomous Region, China (black dot) and the doubtful distribution of this species in Jiangkou County, Guizhou Province, China (black diamond).

*Buforubroventromaculatus* is confirmed from central and northern Vietnam and Wenshan Prefecture in Yunnan Province and Baise City in Guangxi Autonomous Region, China. The distribution of this species in Guizhou Province, China, is still in doubt (Fig. 3).

## Analysis

Morphological measurements of the specimens from China are presented in Table 1. The head width is greater than the head length in all the specimens from China (HW/HL 1.17–1.35), while the head width is smaller than the head length in some individuals of the type series of *Buforubroventromaculatus* (HW/HL 0.88–1.15). Apart from this, there is no significant morphological difference between the specimens from China and the type specimens of *B.rubroventromaculatus* from Vietnam (Table 2).

**Figure 4.**
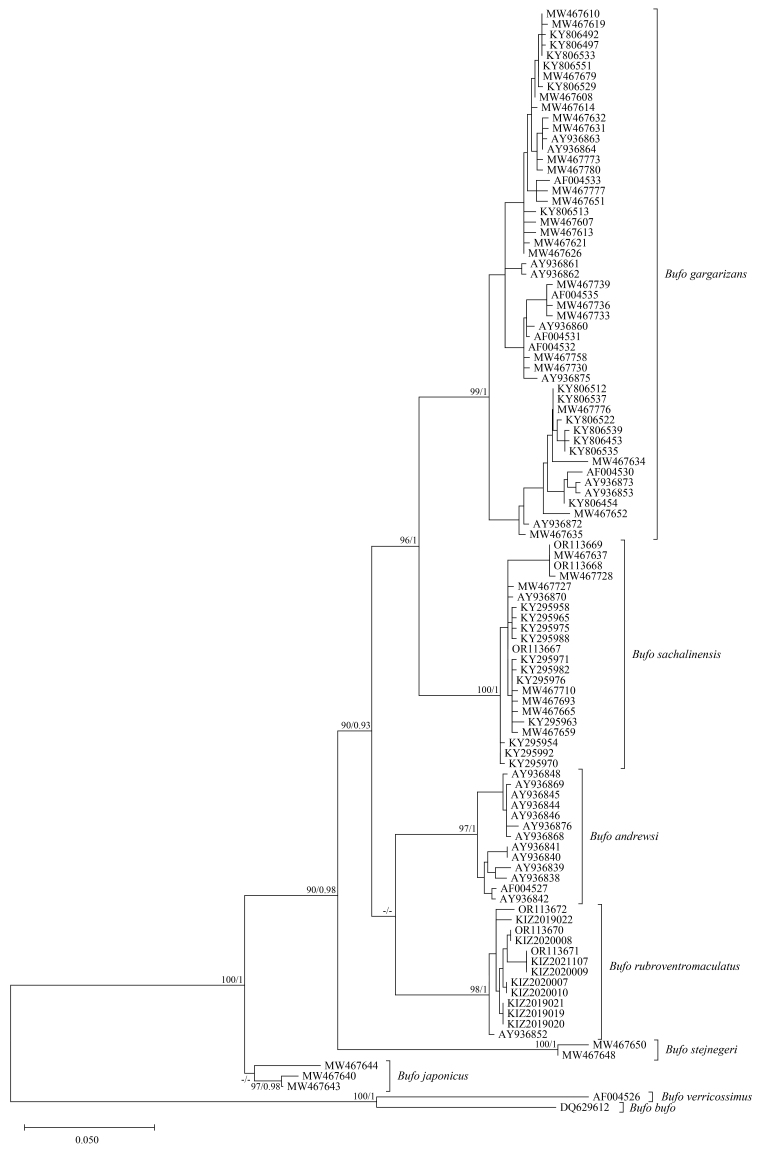
Maximum Likelihood phylogenetic tree, based on ND2 sequences. Numbers after and behind the “/” are Maximum Likelihood ultrafast bootstrap values and Bayesian posterior probabilities, respectively. -/- represents less than 90/0.90.

Maximum Likelihood analysis and Bayesian Inference result in similar topologies. The specimens from China clustered with *Buforubroventromaculatus* from Vietnam (including the holotype) (Fig. 4). The genetic distances (uncorrected p-distance) between the specimens from China and *B.rubroventromaculatus* from Vietnam (including the holotype) ranged from 0 to 1.5% (Table 3).

## Discussion

In Orlov et al. (2024), the description of *Buforubroventromaculatus* is very brief, so it is necessary to make a supplementary description of the species. In this study, we confirmed the distribution of this species in China and provided a more detailed description of this species, based on the specimens collected from China.

According to the original description of *Buforubroventromaculatus*, the head width is smaller than the head length in the female holotype and the male paratype, while the head width is greater than the head length in the female paratype (Orlov et al. 2024). However, the head width is greater than the head length in all the specimens we collected from China. Orlov et al. (2024) provided photos of the holotype with a ruler. According to the photos of the holotype and the ruler, it can be seen that the value of the head length is clearly incorrect and the head width is obviously greater than the head length. Therefore, we consider that the head width should be greater than the head length in females of this species. As for males, we consider that it should be the same. Since Orlov et al. (2024) did not provide any photos for the male paratype, it needs to be verified by re-measurement of the specimen.

In the phylogenetic analysis of Orlov et al. (2024), the sequence (GenBank accession AY936852) of a specimen from Jiangkou County, Guizhou Province, China, clustered with the sequences of *Buforubroventromaculatus*. Our analysis confirmed that this specimen should indeed be assigned to *B.rubroventromaculatus*. However, Jiangkou is far away from the confirmed distribution of *B.rubroventromaculatus* and there is no other record of this species from the area between Jiangkou and the confirmed distribution of this species. Therefore, we are only certain that this species is distributed in Yunnan and Guangxi in China and whether this species is distributed in Guizhou remains to be verified.

## Supplementary Material

XML Treatment for Bufo rubroventromaculatus

## Figures and Tables

**Table 1.** Measurements (in mm) of the specimens of *Buforubroventromaculatus* from China (for abbreviations, see Material and methods).

	KIZ2019019 ♀	KIZ2019020 ♀	KIZ2019021 ♀	KIZ2019022 ♀	KIZ2020007 ♀	KIZ2020008 ♀	KIZ2020009 ♀	KIZ2020010 ♂	KIZ2021107 ♀
SVL	110.7	116.6	123.7	111.0	105.2	103.2	95.5	71.6	99.1
HL	36.5	38.4	39.0	36.7	35.2	35.7	31.9	24.3	34.7
HW	45.9	48.7	50.5	45.1	47.6	45.2	41.3	28.5	43.6
MN	34.3	35.8	35.9	33.4	33.0	32.8	30.2	22.0	31.4
MFE	27.2	29.2	28.3	27.1	27.0	26.7	24.8	17.3	26.2
MBE	21.2	21.6	22.1	21.1	20.4	21.6	18.9	12.6	18.7
SNL	14.5	13.9	15.7	14.7	13.3	14.1	13.2	9.8	13.9
ED	8.9	11.9	9.3	9.1	8.9	9.1	8.7	7.2	9.3
IN	7.3	7.3	7.2	7.2	7.6	7.9	7.2	5.2	7.1
DAE	18.9	17.7	18.7	18.3	17.6	18.4	17.4	13.0	18.6
DPE	29.7	29.9	29.7	28.1	27.6	27.1	26.0	19.1	27.0
NS	7.2	7.4	8.3	7.7	7.2	7.8	6.5	4.9	6.6
EN	8.3	7.1	8.6	7.6	7.3	7.4	7.1	5.7	6.8
FLL	23.4	25.8	27.7	26.4	19.2	20.9	18.2	19.2	23.3
HAL	57.9	57.7	64.4	57.7	54.5	54.7	50.6	40.6	53.9
IPT	4.9	3.6	5.7	5.2	4.8	4.7	4.2	3.3	5.6
OPT	7.0	7.1	7.7	6.9	6.3	6.7	5.7	4.2	5.7
F1	10.8	12.5	14.1	12.6	11.2	11.9	11.4	7.4	11.7
F2	10.6	11.7	13.9	11.5	10.2	10.9	10.6	6.7	11.1
F3	16.9	16.8	18.9	16.2	15.1	15.5	14.5	10.7	15.7
F4	10.9	11.9	14.2	11.9	10.5	11.1	10.8	7.1	11.5

**Table 2.** Comparison between the specimens of *Buforubroventromaculatus* from China and the type series of *B.rubroventromaculatus* from Vietnam (for abbreviations, see Material and methods). Data for the type series of *B.rubroventromaculatus* was obtained from Orlov et al. (2024).

	China ♀♀ (n = 8)	China ♂ (n = 1)	Vietnam ♀♀ (n = 2)	Vietnam ♂ (n = 1)
HL/SVL	0.32–0.35	0.34	0.34–0.52	0.36
HW/HL	1.23–1.35	1.17	0.92–1.15	0.88
SNL/SVL	0.12–0.14	0.14	0.11–0.16	0.16
ED/SVL	0.08–0.10	0.10	0.08–0.11	0.12
DPE/DAE	1.45–1.69	1.47	1.46–1.54	1.43
ED/SNL	0.61–0.86	0.73	0.68–0.77	0.71
IN/SNL	0.46–0.57	0.53	0.49–0.62	0.52
NS/SNL	0.47–0.55	0.50	0.46–0.49	0.48
EN/SNL	0.49–0.57	0.58	0.53–0.57	0.56
FLL/SVL	0.18–0.24	0.27	0.19–0.26	0.28
HAL/SVL	0.49–0.54	0.57	0.48–0.53	0.58
Relative finger length	F3 > F1 > F4 > F2 or F3 > F4 > F1 > F2	F3 > F1 > F4 > F2	F3 > F1 > F4 > F2 or F3 > F4 > F1 > F2	F3 > F4 > F1 > F2

**Table 3.** Uncorrected pairwise genetic distances (%) with the sequences of *Buforubroventromaculatus*.

	1	2	3	4	5	6	7	8	9	10	11	12
1 KIZ2019019												
2 KIZ2019020	0.0											
3 KIZ2019021	0.0	0.0										
4 KIZ2019022	0.7	0.7	0.7									
5 KIZ2020007	0.4	0.4	0.4	0.8								
6 KIZ2020008	0.5	0.5	0.5	1.0	0.4							
7 KIZ2020009	1.0	1.0	1.0	1.4	0.8	0.7						
8 KIZ2020010	0.4	0.4	0.4	0.8	0.0	0.4	0.8					
9 KIZ2021107	1.0	1.0	1.0	1.5	0.8	0.7	0.0	0.8				
10 OR113670	0.6	0.6	0.6	0.9	0.6	0.0	0.7	0.6	0.7			
11 OR113671	0.9	0.9	0.9	1.3	0.9	0.7	0.0	0.9	0.0	0.7		
12 OR113672	0.9	0.9	0.9	0.9	0.9	1.1	1.5	0.9	1.5	1.1	1.5	
13 AY936852	0.6	0.6	0.6	0.9	0.8	0.9	1.4	0.8	1.4	0.7	1.1	0.7

## References

[B11944213] Burland Timothy (2000). DNASTAR's Lasergene Sequence Analysis Software. Methods in Molecular Biology.

[B11944230] Edler Daniel, Klein Johannes, Antonelli Alexandre, Silvestro Daniele (2021). RaxmlGUI 2.0: A graphical interface and toolkit for phylogenetic analyses using RAxML. Methods in Ecology and Evolution.

[B11944239] Frost D Amphibian species of the world: an online reference. Version 6.1. Electronic Database. http://research.amnh.org/herpetology/amphibia/index.html.

[B11944263] Fu Jinzhong, Weadick Cameron, Zeng Xiaomao, Wang Yuezhao, Liu Zhijun, Zheng Yuchi, Li Cheng, Hu Ying (2005). Phylogeographic analysis of the *Bufogargarizans* species complex: A revisit. Molecular Phylogenetics and Evolution.

[B11944276] Kalyaanamoorthy Subha, Minh Bui, Wong Thomas, von Haeseler Arndt, Jermiin Lars (2017). ModelFinder: fast model selection for accurate phylogenetic estimates. Nature Methods.

[B11944286] Macey J, Schulte James, Larson Allan, Fang Zhili, Wang Yeuzhao, Tuniyev Boris, Papenfuss Theodore (1998). Phylogenetic relationships of toads in the *Bufobufo* species group from the Eastern Escarpment of the Tibetan Plateau: A case of vicariance and dispersal. Molecular Phylogenetics and Evolution.

[B11944298] Orlov Nikolai, Ananjeva Natalia, Ermakov Oleg, Lukonina Svetlana, Ninh Hoa, Nguyen Tao (2024). A new record of *Bufogargarizans* complex (Bufonidae, Anura) from Truong Son Mounts, Ha Tinh and Ha Giang Provinces, Vietnam based on molecular evidence with a description of a new species. Diversity.

[B11944317] Ronquist Fredrik, Teslenko Maxim, van der Mark Paul, Ayres Daniel, Darling Aaron, Höhna Sebastian, Larget Bret, Liu Liang, Suchard Marc, Huelsenbeck John (2012). MrBayes 3.2: Efficient Bayesian phylogenetic inference and model choice across a large model space. Systematic Biology.

[B11944332] Tamura Koichiro, Stecher Glen, Kumar Sudhir (2021). MEGA11: Molecular Evolutionary Genetics Analysis Version 11. Molecular Biology and Evolution.

[B11944341] Thompson Julie, Gibson Toby, Higgins Des (2002). Multiple Sequence Alignment Using ClustalW and ClustalX. Current Protocols in Bioinformatics.

